# A Primer of Psychology and Mental Disease

**Published:** 1907-04

**Authors:** 


**A Primer of Psychology and Mental Disease.** For use in Training Schools for Attendants and Nurses and in Medical Classes, and as a ready reference for the practitioner. By **C. B. Burr,** M.D., Medical Director of Oak Grove Hospital (Flint, Mich.) for Mental and Nervous Diseases. Third edition. Revised, with illustrations. Pages viii-183, 12mo. Philadelphia: F. A. Davis Company, (Price, $1.25 net).

This is a very handy and readable book and in spite of its primary size and presentation of the elements of the subject, it contains all the material necessary for a fairly solid ground work for the further study of a most fascinating subject. It is clear and comprehensive and covers the ground of elementary study completely. After a careful reading of the primer a man who could be guilty of a Wileyism would be unforgivable. In this edition the forms of disease have been rewritten to bring the book into accord with the newer classification of insanities.

